# The impact of preoperative handgrip strength on postoperative outcomes following transforaminal lumbar interbody fusion

**DOI:** 10.1186/s13018-025-05717-z

**Published:** 2025-03-28

**Authors:** Duy Nguyen Anh Tran, Yu-Pin Chen, Hui-En Lin, Tan Thanh Nguyen, Hoan Le Nguyen, Yi-Jie Kuo

**Affiliations:** 1 The International PhD Program in Medicine, College of Medicine, Taipei Medical University, Taipei, Taiwan; 2 Department of Orthopedics, Faculty of Medicine, Can Tho University of Medicine and Pharmacy, Can Tho, Vietnam; 3 Department of Orthopedic surgery, Wan-Fang Hospital, Taipei Medical University, Taipei, Taiwan; 4 Department of Orthopedic surgery, School of Medicine, College of Medicine, Taipei Medical University, Taipei, Taiwan

**Keywords:** Handgrip strength, Transforaminal lumbar interbody fusion, JOA, EQ-5D-3L, Barthel index, Spine surgery

## Abstract

**Background:**

With an aging population, the prevalence of lumbar spinal diseases necessitating surgical intervention is increasing. Handgrip strength (HGS) has emerged as a simple measure of muscle function that may correlate with surgical outcomes. However, the role of HGS concerning postoperative recovery following transforaminal lumbar interbody fusion (TLIF) is not well-studied, highlighting a gap in the literature regarding its potential as a prognostic tool.

**Methods:**

This prospective observational study included 89 patients who underwent TLIF performed by a single surgeon. Patients were categorized into normal and low HGS groups based on preoperative HGS measurements. Demographics, baseline HGS, and surgical details were recorded, and outcomes were assessed using the JOA, EQ-5D-3L, and Barthel Index at 3, 6, and 12 months postoperatively. Generalized Estimating Equations were used to examine associations between baseline parameters and outcomes over time.

**Results:**

All patients were followed for at least one year, except for 15 (15.6%) who were lost to follow-up before the one-year mark. Patients with lower preoperative HGS were associated with significantly poorer postoperative functional outcomes. Specifically, a one-unit decrease in HGS was associated with a 2.551-point decrease in the JOA score (*p* = 0.008), a 0.142-point decrease in the EQ-5D-3L score (*p* = 0.007), and a 5.784-point decrease in the Barthel Index (*p* = 0.036). Additionally, male sex, higher body mass index, and lower Charlson comorbidity index were associated with better postoperative outcomes.

**Conclusions:**

Low preoperative handgrip strength is associated with poorer functional, quality of life, and independence outcomes up to 12 months after TLIF surgery. Assessing HGS preoperatively may provide clinicians with valuable information for identifying patients at risk of suboptimal recovery. Future research could explore intervention strategies to improve preoperative muscle function and potentially enhance recovery outcomes for patients undergoing TLIF.

**Supplementary Information:**

The online version contains supplementary material available at 10.1186/s13018-025-05717-z.

## Introduction

As lifespans lengthen, a rise in age-related spinal disorders, particularly in the lumbar region, presents a growing challenge [1, 2]. These conditions are often attributed to the degeneration and weakening of bones, discs, and surrounding soft tissues [3]. Transforaminal lumbar interbody fusion (TLIF) surgery is a well-established and effective treatment option for various lumbar spinal issues [4]. TLIF is a spinal fusion procedure that can be performed minimally invasively, involving the removal of the intervertebral disc followed by the insertion of an implant to stabilize the spine [4–6]. This process aims to foster bone growth (osteogenesis) and eventually fuse two or more vertebrae, with the goal of reducing pain and enhancing functionality [4, 5, 7].

Handgrip strength (HGS), a convenient measure of voluntary muscle function, has become recognized as a significant biomarker of our health [8]. Its ease of use, speed, low cost, and simplicity make it a valuable tool. Increasingly, research underscores the strong predictive power of HGS for assessing nutritional status and sarcopenia (muscle loss) [9, 10]. While sarcopenia, as defined by the Asian Working Group for Sarcopenia (AWGS) 2019 [10], is diagnosed based on a combination of low muscle strength, low muscle quantity/quality, and low physical performance, its assessment may pose challenges in patients with lumbar spine degeneration. Specifically, physical performance metrics, such as gait speed or timed-up-and-go tests, could be influenced by preexisting spinal pathology, potentially introducing bias into the evaluation process. In contrast, HGS offers a practical and accessible alternative, as it directly measures muscle function without being significantly affected by lumbar spine degeneration. This study, therefore, focuses on HGS as a straightforward, reliable indicator to investigate its association with postoperative outcomes following TLIF.

Although previous studies have demonstrated a correlation between HGS and overall outcomes following spine surgery [11–13], the predictive value of baseline HGS on specific postoperative outcomes in patients undergoing TLIF remains unclear. This study aims to determine whether preoperative HGS is associated with postoperative functional outcomes, quality of life, and independence in activities of daily living at one-year post-TLIF. To account for the longitudinal nature of the data and potential associations among repeated measurements, we will employ a generalized estimating equation (GEE) model [14, 15]. We hypothesize that higher baseline HGS will be associated with more favorable outcomes.

## Methods

### Study design and setting

This prospective observational study recruited patients from a single hospital between January 2020 and June 2023. A total of 103 consecutive patients were scheduled for TLIF surgery based on surgical indications and contraindications described in previous studies [5, 16]. Patients aged 18 years or older with TLIF indications such as lumbar spinal stenosis, lumbar disc herniation, or low-grade lumbar spondylolisthesis (Meyerding I or II) were included. Diagnosis was confirmed using standing radiographs and MRI, and all patients must have experienced lower back and radiating pain without improvement after at least three months of conservative treatment. Exclusion criteria comprised patients with previous lumbar spine surgery or revision, high-grade spondylolisthesis (Meyerding > II), cervical stenosis (tandem stenosis), degenerative scoliosis, and those diagnosed or suspected to have underlying or ongoing diseases such as spondylodiscitis, ankylosing spondylitis, spinal neoplasm, spinal metastasis, and traumatic spine injury. Additionally, individuals diagnosed with cognitive or psychological disorders were excluded. The presence of cervical stenosis was specifically assessed through MRI to prevent any potential confounding effect on handgrip strength measurements. The study adhered to the Declaration of Helsinki and followed the STROBE guidelines for observational studies, with approval from our institute’s Ethics Committee.

Data on basic demographics and health metrics were collected, including age, sex, body mass index (BMI), Charlson comorbidity index (CCI), HGS, bone mineral density (T-score), American Society of Anesthesiologists (ASA) physical status classification, fusion levels, and total intraoperative blood loss.

### HGS measurement and group allocation

Preoperative maximum handgrip strength was assessed in each patient using a Jamar Hydraulic Dynamometer (Sammons Preston, USA), following standardized testing protocols. Patients were seated comfortably in a chair or bed, ensuring their feet were flat on the ground for stability. The test was conducted with the elbow flexed at 90 degrees, the shoulder adducted, and the forearm in a neutral position (mid-pronation). The wrist was kept in a neutral position without extension or flexion to prevent bias from wrist positioning.

Each patient was given clear verbal instructions and a demonstration before performing the test. They were instructed to squeeze the dynamometer as hard as possible for 3–5 seconds, ensuring maximum effort. Three consecutive trials were performed for each hand, with a 30-second rest period between trials to minimize muscle fatigue [17]. The highest recorded value across the three attempts was used for analysis, as recommended by the Asian Working Group for Sarcopenia (AWGS) 2019 guidelines [10].

To ensure consistency and accuracy, all assessments were performed by a trained examiner using the same dynamometer throughout the study. Patients were encouraged with standardized verbal prompts, such as “Squeeze as hard as you can!” to maintain motivation and effort. Any discomfort or pain was noted, but patients experiencing acute hand pain or neuromuscular disorders affecting grip strength were excluded from the study. The final HGS values were categorized based on AWGS criteria, defining low HGS as < 28 kg for males and < 18 kg for females.

### Operative techniques

All surgeries were performed by a single surgeon from the author’s group, using the TLIF techniques. Patients were positioned prone for TLIF procedures. A paramedian incision was utilized for TLIF. Pedicle screws were inserted bilaterally in this procedure. In TLIF, the facet joint was removed to access the disc space, followed by a partial discectomy. All patients received artificial bone grafts using Rafugen™ DBM (Cellumed Co., Ltd., Seoul, Korea) and CeraMatrix bone graft substitute (Xelite Biomed Ltd., Taiwan). The bone graft was packed into the interbody space along with an interbody cage in all patients. Compression of bone grafts, screw head tightening, and placement of a negative pressure drain were performed before wound closure. Postoperatively, all patients received antibiotics, painkillers, and neurotrophic drugs. Drainage tubes were removed at 48 h based on clinical assessment. Patients initiated brace use three days postoperatively for a duration of three months. Strenuous physical activity was restricted throughout this period [5, 18].

### Outcome assessment

Clinical outcomes, including the Japanese Orthopaedic Association (JOA) score and quality of life, were assessed by an independent, blinded investigator at baseline and 3, 6, and 12 months postoperatively.

The JOA score is a validated measure of functional outcome following TLIF [19]. It assesses subjective symptoms (9 points), clinical signs (6 points), and limitations in daily activities (14 points), yielding a total score of 29. A higher JOA score correlates with improved function and reduced pain.

Quality of life was assessed using the EuroQol 5-Dimensions 3-Level version (EQ-5D-3L) questionnaire with quality weights estimated for Taiwan [20]. Higher EQ-5D-3L scores represent a better quality of life.

The Barthel index is a widely used assessment tool to measure independence in activities of daily living [21]. It quantifies a patient’s ability to perform self-care tasks, such as feeding, bathing, dressing, toileting, transferring, continence, mobility, and stair climbing. A higher Barthel index score indicates greater independence and a lower level of disability.

### Statistical analysis

A sample size of 48 participants was determined to be necessary for this study. This calculation was based on a correlation coefficient formula: $\:N=\:{\left(\frac{{Z}_{\alpha\:}+\:{Z}_{\beta\:}}{C}\right)}^{2}+3$ [22], targeting a statistical power of 0.8, a type I error rate of 0.05, and a two-tailed test. The expected correlation coefficient of 0.395, as reported by Kwon O. et al. [13], was utilized to estimate the required sample size.

Statistical analysis was conducted using SPSS (version 30; IBM, Armonk, NY, USA). Descriptive statistics were used, including sample size or frequency (n) with percentages (%) for categorical variables and means ± standard deviations (SD) for continuous variables. Comparisons between groups classified according to HGS values were conducted using appropriate statistical tests. Categorical variables were analyzed using the chi-square or Fisher’s exact test of independence. Continuous variables were analyzed using the Student’s t-test for normally distributed data or the Mann-Whitney U test for non-normally distributed data. A Wilcoxon signed-rank test was conducted to compare the differences between JOA, EQ-5D-3L, and Barthel index assessment time points compared with the baseline. The GEE model was used to assess the effects of various factors on outcomes, accounting for repeated measures within participants over time and providing a robust approach to handling missing data [14, 15]. Sensitivity analyses were conducted to assess the findings’ robustness by modifying the GEE model’s correlation structures and by sequentially adding or removing covariates (e.g., BMI, gender, and CCI) to evaluate their impact on the associations between HGS and postoperative outcomes. Two-sided *p*-values of < 0.05 were considered statistically significant for all tests.

## Results

### Study population selection and patient demographics

**Fig. 1:**
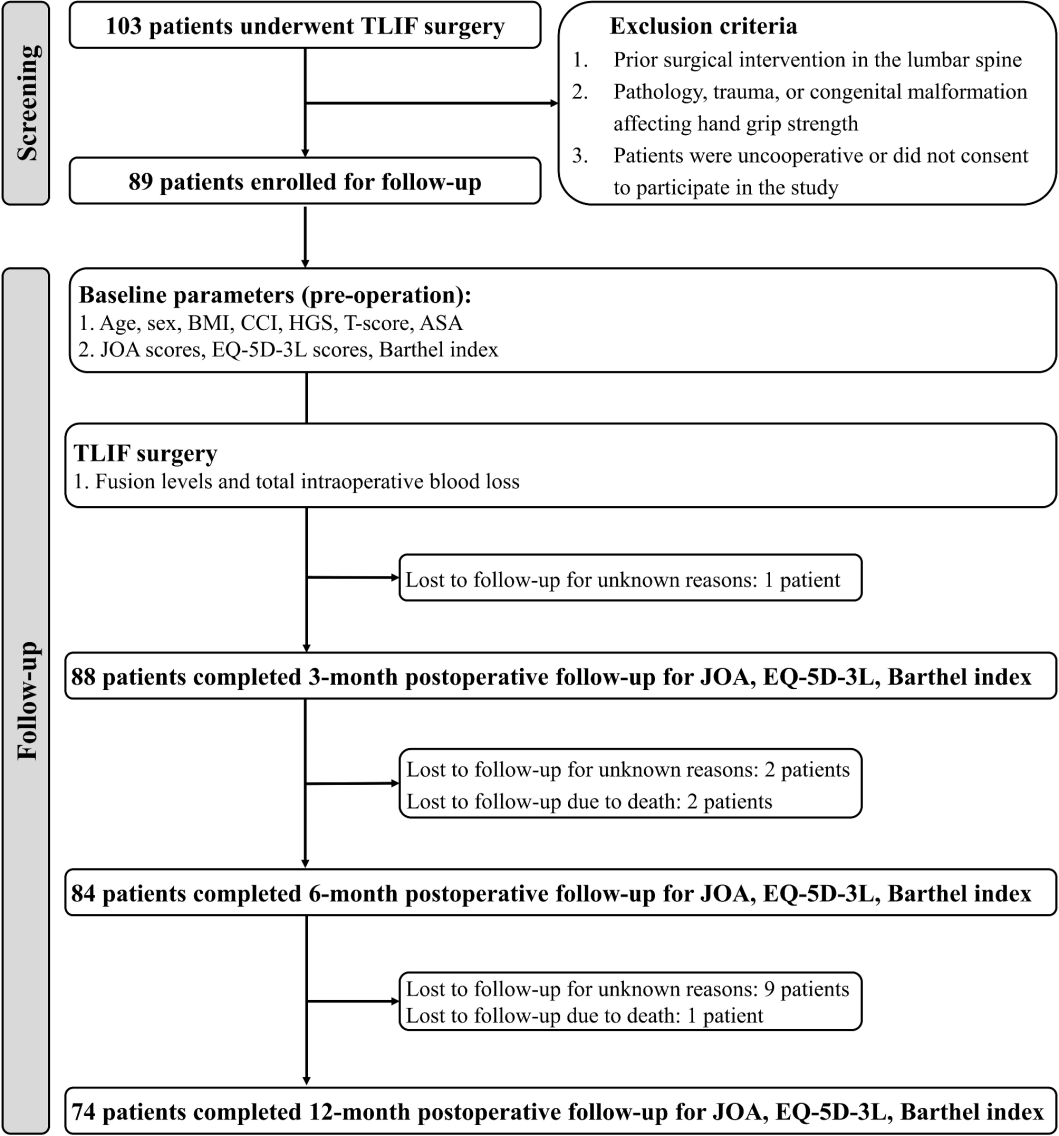
Flowchart of patient selection and follow-up. Abbreviations: TLIF, transforaminal lumbar interbody fusion; BMI, Body mass index; CCI, Charlson comorbidity index; HCG, Hand grip strength; ASA, American Society of Anesthesiologists; JOA, Japanese Orthopaedic Association; EQ-5D-3L, EuroQol 5-Dimensions 3-Level version

Out of the 103 patients who underwent TLIF, 89 were enrolled for follow-up. By the 3-month mark, 88 patients completed the follow-up, with one lost to follow-up for unknown reasons. Between 3 and 6 months, an additional two patients were lost to follow-up, and another two passed away, reducing the number of evaluable patients to 84 at 6 months. Unfortunately, by the final 1-year follow-up, the number of patients had further declined, with only 74 completing the evaluation due to the loss of nine more patients and two additional deaths (Fig. 1).

Of the 89 patients, 46 exhibited normal HGS, and 43 presented with low HGS. The average age of the normal HGS group (65.78 ± 9.80) was significantly younger than that of the low HGS group (72.07 ± 8.40 years, t = -3.239, *p* = 0.002). The sex distribution between the two HGS groups was not statistically significant (*p* = 0.135). The normal HGS group had 45.7% males and 54.3% females, while the low HGS group had 30.2% males and 69.8% females. In addition, patients with normal HGS had significantly higher BMI than those with low HGS (Z = -2.681, *p* = 0.007). Furthermore, a low T-score was associated with low HGS (Z = -2.290, *p* = 0.022). Other variables, including the CCI, incidence of osteoporosis, spondylolisthesis, number of fusion levels, ASA classification, surgical time, and total intraoperative blood loss, were detailed in Table 1. However, no significant differences were found between the normal and low HGS groups for these factors.

**Table 1:** Descriptive statistics of the patients

Parameters	Overall (*n* = 89)	Handgrip strength groups			
		Normal HGS (*n* = 46)	Low HGS (*n* = 43)	*p*	
**Age**,** mean ± SD**	68.82 ± 9.63	65.78 ± 9.80	72.07 ± 8.40	**0.002**	
**Sex**					
**Male**,** n (%)**	34 (38.2)	21 (45.7)	13 (30.2)	0.135	
**Female**,** n (%)**	55 (61.8)	25 (54.3)	30 (69.8)		
**BMI**,** mean ± SD**	25.82 ± 9.63	27.13 ± 4.66	24.41 ± 4.11	**0.007**	
**Underweight**,** n (%)**	2 (22.0)	0	2 (4.7)	0.062	
**Normal**,** n (%)**	44 (49.4)	19 (41.3)	25 (58.1)		
**Overweight**,** n (%)**	24 (27.0)	13 (28.3)	11 (25.6)		
**Obesity**,** n (%)**	19 (21.3)	14 (30.4)	5 (11.6)		
**CCI**,** mean ± SD**	0.82 ± 1.28	0.67 ± 1.08	0.98 ± 1.46	0.330	
**T-score**,** mean ± SD**	-2.23 ± 1.26	-2.03 ± 1.00	-2.43 ± 1.45	**0.022**	
**Osteoporosis**					
**Normal**,** n (%)**	5 (6.2)	3 (7.7)	2 (4.8)	0.141	
**Low bone mass**,** n (%)**	38 (46.9)	20 (51.3)	18 (42.9)		
**Osteoporosis**,** n (%)**	38 (46.9)	16 (41.0)	22 (52.4)		
**Spondylolisthesis**,** n (%)**	43 (48.3)	22 (47.8)	21 (48.8)	0.924	
**Levels of fusion**					
**1**,** n (%)**	61 (68.5)	33 (71.7)	28 (65.1)		
**2**,** n (%)**	24 (27.0)	11 (23.9)	13 (30.2)	0.859	
**3**,** n (%)**	4 (4.5)	2 (4.3)	2 (4.7)		
**ASA**					
**1**,** n (%)**	6 (6.7)	3 (6.5)	3 (7.0)		
**2**,** n (%)**	69 (77.5)	38 (82.6)	31 (72.1)	0.432	
**3**,** n (%)**	14 (15.7)	5 (10.9)	9 (20.9)		
**Surgical time (minutes)**,** mean ± SD**	194.38 ± 77.09	204.54 ± 92.23	183.51 ± 55.69	0.393	
**Blood loss (ml)**,** mean ± SD**	239.09 ± 287.78	276.00 ± 290.08	200.47 ± 283.57	0.058	
**Lost follow-up at 12 months**,** n (%)**	15 (16.9)	10 (21.7)	5 (11.6)	0.203	

**Abbreviations**: HSG, Handgrip strength; BMI, body mass index; CCI, Charlson Comorbidity Index; ASA, American Society of Anesthesiologists; n, number; SD, standard deviations.

**Note**: Cut-off values for HGS were less than 26 kg for men and less than 18 kg for women. Statistically significant values are bolded.

### Surgical outcome analysis

**Fig. 2:**
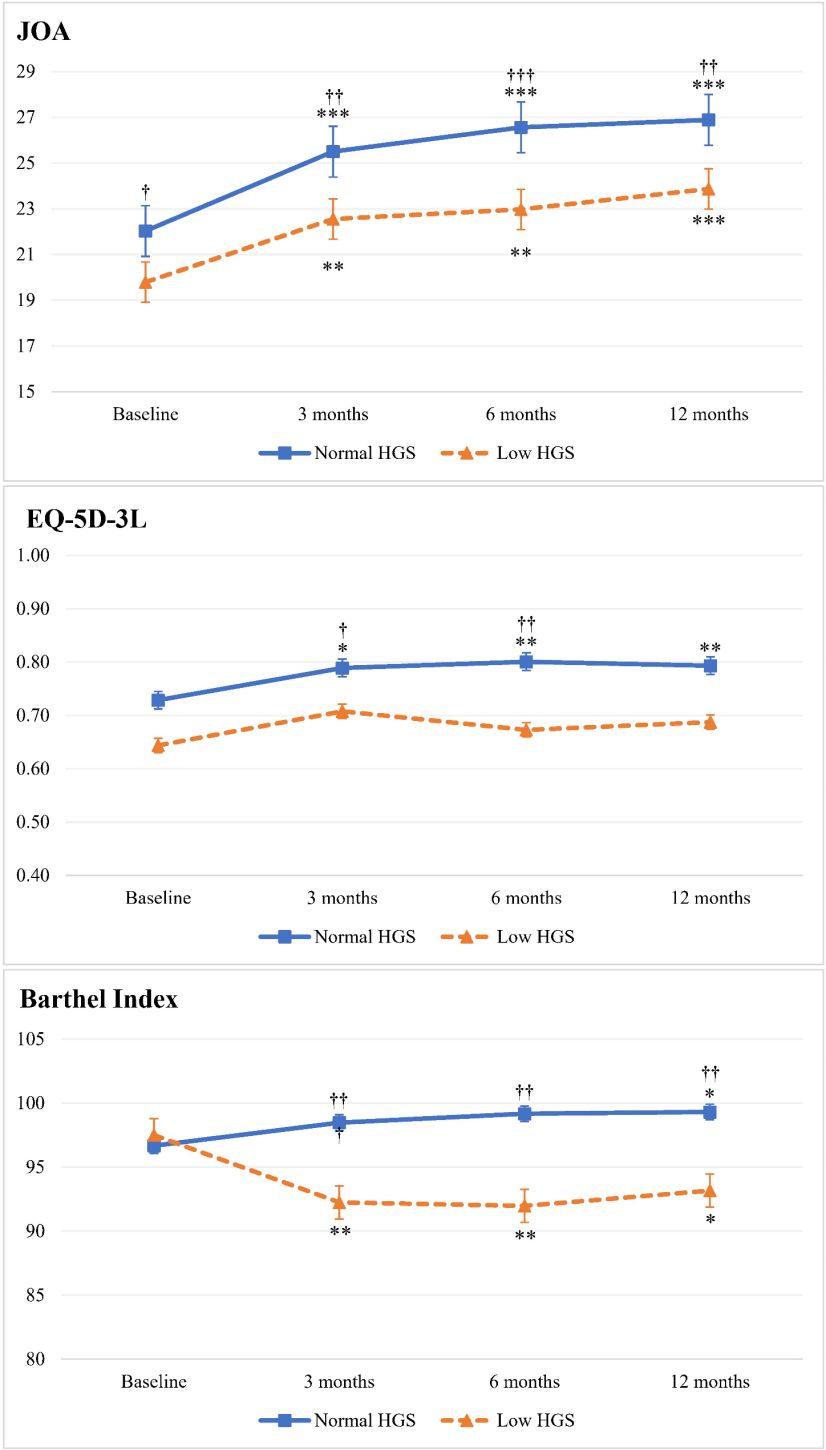
Line graph with whiskers illustrating changes in postoperative outcomes between the two HGS groups. *p < 0.05, **p < 0.01, and ***p < 0.001 indicate statistically significant differences compared to baseline. ^†^*p* < 0.05, ^††^*p* < 0.01, and ^†††^*p* < 0.001 denote statistically significant differences between normal and low HGS groups

JOA scores significantly improved in both HGS groups at 3, 6, and 12 months postoperatively compared to baseline (*p* < 0.001, Fig. 2). Notably, the normal HGS group consistently demonstrated significantly higher JOA scores than the low HGS group at all time points (*p* = 0.036, *p* = 0.006, *p* < 0.001, *p* = 0.001 for baseline, 3 months, 6 months, and 12 months postoperation, respectively). Regarding EQ-5D-3L scores, significant improvements were observed only in the normal HGS group over the 12 months postoperatively (*p* = 0.015, *p* = 0.007, *p* = 0.003 for 3 months, 6 months, and 12 months, respectively). Additionally, at 3 and 6 months, the normal HGS group showed a significant increase compared to the low HGS group, with *p*-values of 0.024 and 0.003, respectively. The Barthel Index revealed a statistically significant improvement in the normal HGS group after 12 months (*p* = 0.035), while the low HGS group experienced a significant decline at all follow-up time points (*p* = 0.001, *p* = 0.004, *p* = 0.017 for 3 months, 6 months, and 12 months, respectively). Furthermore, statistically significant differences were noted between the normal and low HGS groups at 3 months (*p* = 0.001), 6 months (*p* = 0.001), and 12 months (*p* = 0.006).

### Associations of baseline parameters with outcome measurements

To examine the associations between the parameters listed in Table 1 and the outcome variables over time, we employed a GEE model. The results of this analysis are presented in Table 2.

**Table 2:** GEE model of baseline parameters associated with postoperative outcome measurements

Parameters	JOA			EQ-5D-3L			Barthel index		
	β	SE	*p*	β	SE	*p*	β	SE	*p*
Age	0.091	0.0572	0.114	0.006	0.0034	0.078	0.072	0.1626	0.659
Male (ref: female)	**11.840**	**3.3015**	**< 0.001**	**0.344**	**0.1684**	**0.041**	**17.026**	**8.5286**	**0.046**
BMI (ref: Obesity)	**5.044**	**1.3288**	**< 0.001**	**0.161**	**0.0688**	**0.019**	**7.240**	**3.4619**	**0.036**
Underweight	1.629	1.6513	0.324	**0.246**	**0.1075**	**0.022**	3.208	4.8521	0.509
Normal	-0.153	1.0699	0.886	-0.017	0.0531	0.753	0.558	2.3454	0.812
Overweight	-1.568	1.4533	0.281	-0.099	0.0710	0.164	-3.600	4.1731	0.388
CCI	**-1.104**	**0.3599**	**0.002**	-0.028	0.0226	0.216	-0.901	0.9878	0.362
T-score (ref: Osteoporosis)	0.242	0.3797	0.524	0.042	0.0299	0.158	1.045	1.1945	0.381
Normal	1.017	1.9484	0.602	-0.140	0.1294	0.278	-0.551	4.9836	0.912
Low bone mass	0.483	1.1103	0.664	-0.006	0.0623	0.923	-0.260	2.6217	0.921
Spondylolisthesis	0.807	0.9214	0.381	0.002	0.0494	0.962	2.267	2.1974	0.302
Fusion levels (ref: 3-level)									
1-level	-3.287	2.1039	0.118	0.129	0.1076	0.230	-1.248	8.4883	0.883
2-level	-1.211	1.8963	523	0.174	0.0957	0.068	-1.095	7.4262	0.883
Low HGS (ref: Normal HGS)	**-2.551**	**0.9651**	**0.008**	**-0.142**	**0.0522**	**0.007**	**-5.784**	**2.7598**	**0.036**
ASA (ref: ASA 3)									
1	1.709	1.7815	0.338	0.126	0.0996	0.206	4.877	4.5370	0.282
2	-0.312	1.0828	0.74	-0.071	0.0677	0.296	-0.793	3.0051	0.792
Operation time	-0.002	0.0082	0.768	3.11E-5	0.0003	0.919	0.015	0.0280	0.580
Blood loss	-0.002	0.0016	0.126	1.70E-4	8.99E-5	0.059	-0.004	0.0060	0.501
Time (ref: Baseline)									
3 months	**2.739**	**0.6436**	**< 0.001**	**0.060**	**0.0254**	**0.018**	**-4.728**	**2.0473**	**0.021**
6 months	**3.753**	**0.7018**	**< 0.001**	**0.072**	**0.0248**	**0.004**	-3.972	2.0754	0.056
12 months	**4.536**	**0.6100**	**< 0.001**	**0.086**	**0.0284**	**0.003**	-2.608	1.8876	0.167

**Abbreviations**: JOA, Japanese Orthopaedic Association; EQ-5D-3L, EuroQol 5-Dimensions 3-Level; HSG, Handgrip strength; BMI, body mass index; CCI, Charlson comorbidity index; ASA, American Society of Anesthesiologists; β, beta coefficient; SE, standard error; ref, reference.

**Note**: Cut-off values for HGS were less than 26 kg for men and less than 18 kg for women. Statistically significant values are bolded.

### JOA scores

Results indicated that male sex (β = 11.840, *p* < 0.001), increased BMI (β = 5.044, *p* < 0.001), and lower CCI (β = -1.104, *p* = 0.002) were significantly linked with higher JOA scores. Conversely, lower HGS (β = -2.551, *p* = 0.008) was associated with lower JOA scores. Additionally, JOA scores demonstrated significant improvement over time compared to baseline at 3 months (β = 2.739, *p* < 0.001), 6 months (β = 3.753, *p* < 0.001), and 12 months (β = 4.536, *p* < 0.001).

### EQ-5D-3L scores

Consistent with JOA findings, EQ-5D-3L scores were significantly positively connected with male sex (β = 0.344, *p* = 0.041), BMI (β = -0.161, *p* = 0.019), and normal HGS group (β = 0.142, *p* = 0.007). Notably, underweight individuals exhibited significantly higher EQ-5D-3L scores compared to obese individuals (β = 0.246, *p* = 0.022). Postoperative EQ-5D-3L scores were significantly elevated at all time points relative to baseline: 3 months (β = 0.060, *p* = 0.018), 6 months (β = 0.072, *p* = 0.004), and 12 months (β = 0.086, *p* = 0.003).

### Barthel index

A lower Barthel Index was exclusively associated with patients in the low HGS group at 12-month follow-up (β = -5.703, *p* = 0.037). Additionally, male sex (β = 17.026, *p* = 0.046) and higher BMI (β = 7.240, *p* = 0.036) were identified as significant predictors of better functional independence outcomes. In contrast to JOA and EQ-5D-3L, Barthel Index experienced a significant decline after 3 months compared to baseline (β = -4.720, *p* = 0.020).

## Discussion

Effective treatment outcomes require a thorough understanding and management of associated risks. Our study identified sex, BMI, CCI, and particularly HGS as significant predictors of postoperative outcomes, as measured by the JOA score, EQ-5D-3L, and Barthel index.

Our study demonstrates that the Transforaminal Lumbar Interbody Fusion (TLIF) procedure consistently yields significant improvements in functional recovery and quality of life. This is evidenced by substantial enhancements in Japanese Orthopaedic Association (JOA), EuroQol five-dimension three-level (EQ-5D-3L), and Barthel Index scores over time, reinforcing the well-established effectiveness of TLIF in managing lumbar spine disorders through pain alleviation and enhanced functional capacity. Furthermore, postoperative complications were minimal, with only a single patient experiencing a superficial surgical site infection that fully resolved within a week. Notably, advancements in TLIF techniques, such as endoscopic approaches and refined safe operating zone identification, have further contributed to positive patient outcomes and accelerated recovery [6, 23–25]. These findings collectively underscore TLIF as a reliable surgical intervention for optimizing patient outcomes, particularly when coupled with meticulous preoperative assessments and structured postoperative care.

HGS is a critical component in sarcopenia assessment [10]. Our analysis demonstrated a significant association of low baseline HGS with older age, lower BMI, and reduced bone mineral density (Table 1). Notably, while not statistically significant, the female sex ratio was twice as high in the HGS group compared to males, suggesting a potential sex-related influence on HGS. This aligns with previous research demonstrating that although age universally impacts muscle structure and function, females tend to exhibit a higher sarcopenia prevalence at earlier ages than males [26–28], often accompanied by osteoporosis [1, 29]. By contrast, the link between BMI and HGS in the elderly is debated [30]; This ambiguity persists in the context of lumbar spine surgery. While most studies in this area suggest a nonsignificant trend towards higher BMI in individuals with low HGS [11, 13, 31], contradictory findings, such as those reported by F. Shen [12], underscore the complex relationship between these variables. Further investigation is warranted to clarify the interplay between BMI and HGS in this population.

HGS is a recognized predictor of outcomes after various types of surgery [32–35], including lumbar spine surgery. Previous research consistently links low HGS to poorer rehabilitation outcomes, often assessed using the Oswestry disability index (ODI) and EQ-5D [11–13]. To comprehensively assess the impact on daily life, we employed the JOA index, a well-established measure highly correlated with the ODI [18, 30], in conjunction with the EQ-5D and Barthel indices. Our findings reveal significantly greater improvements in all three indices among patients with normal HGS compared to those with low HGS, aligning with previous research. Furthermore, we demonstrated a significant association between low HGS and decreased postoperative outcomes using the GEE model. This finding is consistent with the link between low HGS and various adverse health conditions, such as sarcopenia, poor bone quality, and frailty [8, 36], which can hinder recovery and treatment efficacy. Our results suggest that HGS is valuable for assessing preoperative functional status and predicting lumbar interbody fusion surgery outcomes.

Our analysis revealed that female sex is a significant predictor of poor prognosis following TLIF surgery, a finding consistent with numerous previous studies, including a systematic review [37–39]. In addition to its association with lower JOA and EQ-5D-3L scores, female sex was also significantly correlated with poorer Barthel Index scores at 12 months (β = 17.026, *p* = 0.046), suggesting that men had a greater likelihood of maintaining postoperative functional independence. Researchers have suggested that estrogen deficiency during menopause, resulting in decreased bone quality, is a primary factor contributing to this condition. Additionally, studies have indicated that women generally have a lower pain tolerance than men [40, 41], which may affect the pain-related scores which may influence pain-related scores, which are one of the main criteria of the JOA scale.

Obesity has been linked to increased postoperative complications in spine surgery [42–44], yet its impact on functional outcomes remains controversial [45, 46]. While previous meta-analyses have not identified a consistent association between obesity and functional scores [47, 48], our study suggests a potential positive connection between BMI and postoperative JOA, EQ-5D-3L, and Barthel Index scores. However, a closer examination of BMI subgroups, we did not observe statistically significant associations with these outcomes. In addition, although there was an increase in EQ-5D-3L scores compared with the obesity group, the limited number of patients in the underweight group (*n* = 2) underpowered this finding. Larger studies are needed to clarify this finding.

CCI is a well-established predictor of postoperative JOA improvement rate [49], complications, reoperations, and mortality [50–53] in spine surgery. Our findings corroborate previous research by demonstrating a significant inverse relationship between CCI and both JOA and EQ-5D scores. These results underscore the critical role of comprehensive comorbidity assessment and management in optimizing patient outcomes following spine surgery.

We acknowledge several limitations in this study. The research was conducted at a single center and involved a single surgeon during the COVID-19 pandemic, which may have restricted the sample size and may not represent the diversity of patient populations and surgical practices across different settings. Additionally, approximately 17% of patients were lost to follow-up at 12 months postoperatively for unknown reasons, potentially biasing our final results. Due to significant sex differences in HGS, continuous data analysis was not feasible, leading us to use AWGS criteria for sarcopenia classification– a method that may not be optimal for our population. Moreover, while AWGS 2019 provides widely accepted cut-off values for HGS, these thresholds are fixed and do not account for age-related variations. Future research should consider age-specific thresholds to better assess sarcopenia risk across different age groups. Furthermore, our focus on preoperative HGS alone precludes an assessment of whether postoperative changes in HGS could predict recovery outcomes. Further research involving larger, multicenter studies is necessary to confirm these findings, explore potential interventions to improve outcomes for patients with low HGS, and establish more precise cut-off values for different subgroups, including male and female populations.

## Conclusion

This study demonstrated that preoperative HGS is a significant predictor of postoperative functional outcomes following TLIF surgery. Patients with normal HGS exhibited superior improvements in JOA, EQ-5D-3L, and Barthel Index scores compared to those with low HGS. These findings highlight the importance of preoperative HGS assessment in patient selection and management for TLIF surgery.

## Electronic supplementary material

Below is the link to the electronic supplementary material.

Supplementary Material 1

## Data Availability

No datasets were generated or analysed during the current study.

## Abbreviations

ASA: American society of anesthesiologists
AWGS: Asian working group for sarcopenia
BMI: Body mass index
CCI: Charlson comorbidity index
EQ-5D-3L: EuroQol 5-dimensions 3-level version
GEE: Generalized estimating equation
HGS: Handgrip strength
JOA: Japanese orthopaedic association
ODI: Oswestry disability index
SD: Standard deviations
TLIF: Transforaminal lumbar interbody fusion

## References

[1] Nguyen HT, Nguyen BT, Thai THN, Tran AV, Nguyen TT, Vo T, et al. Prevalence, incidence of and risk factors for vertebral fracture in the community: the Vietnam osteoporosis study. Sci Rep. 2024;14(1):32. 10.1038/s41598-023-50145-w 38168502 10.1038/s41598-023-50145-w PMC10761939

[2] Lang S, Walter N, Freigang V, Neumann C, Loibl M, Alt V, et al. Increased incidence of vertebral fractures in German adults from 2009 to 2019 and the analysis of secondary diagnoses, treatment, costs, and in-hospital mortality. Sci Rep. 2023;13(1):6984. 10.1038/s41598-023-31654-0 37117230 10.1038/s41598-023-31654-0 PMC10147602

[3] Benoist M. Natural history of the aging spine. Eur Spine J. 2003;12(2):S86–9. 10.1007/s00586-003-0593-0 12961079 10.1007/s00586-003-0593-0 PMC3591827

[4] Kim YH, Ha KY, Rhyu KW, Park HY, Cho CH, Kim HC, et al. Lumbar interbody fusion: techniques, pearls and pitfalls. Asian Spine J. 2020;14(5):730–41. 10.31616/asj.2020.0485 33108838 10.31616/asj.2020.0485 PMC7595814

[5] Mobbs RJ, Phan K, Malham G, Seex K, Rao PJ. Lumbar interbody fusion: techniques, indications and comparison of interbody fusion options including PLIF, TLIF, MI-TLIF, OLIF/ATP, LLIF and ALIF. J Spine Surg. 2015;1(1):2–18. 10.3978/j.issn.2414-469X.2015.10.05 27683674 10.3978/j.issn.2414-469X.2015.10.05 PMC5039869

[6] Bahir AW, Daxing W, Jiayu X, Bailian L, Shao G. Comparative efficacy and fusion outcomes of unilateral bi-portal endoscopic transforaminal lumbar interbody fusion versus minimally invasive transforaminal lumbar interbody fusion in treating single-segment degenerative lumbar spondylolisthesis with lumbar spinal stenosis: a two-year retrospective study. J Orthop Surg Res. 2024;19(1):835. 10.1186/s13018-024-05315-5 39696362 10.1186/s13018-024-05315-5 PMC11657107

[7] de Kunder SL, Rijkers K, Caelers I, de Bie RA, Koehler PJ, van Santbrink H. Lumbar interbody fusion: A historical overview and a future perspective. Spine (Phila Pa 1976). 2018;43(16):1161–8. 10.1097/BRS.0000000000002534 29280929 10.1097/BRS.0000000000002534

[8] Vaishya R, Misra A, Vaish A, Ursino N, D’Ambrosi R. Hand grip strength as a proposed new vital sign of health: a narrative review of evidences. J Health Popul Nutr. 2024;43(1):7. 10.1186/s41043-024-00500-y 38195493 10.1186/s41043-024-00500-y PMC10777545

[9] Lee SY. Handgrip strength: an irreplaceable indicator of muscle function. Ann Rehabil Med. 2021;45(3):167–9. 10.5535/arm.21106 34233405 10.5535/arm.21106 PMC8273729

[10] Chen LK, Woo J, Assantachai P, Auyeung TW, Chou MY, Iijima K, et al. Asian working group for sarcopenia: 2019 consensus update on sarcopenia diagnosis and treatment. J Am Med Dir Assoc. 2020;21(3):300. 10.1016/j.jamda.2019.12.012. 7 e2. 32033882 10.1016/j.jamda.2019.12.012

[11] Kwon JW, Lee BH, Lee SB, Sung S, Lee CU, Yang JH, et al. Hand grip strength can predict clinical outcomes and risk of falls after decompression and instrumented posterolateral fusion for lumbar spinal stenosis. Spine J. 2020;20(12):1960–7. 10.1016/j.spinee.2020.06.022 32622937 10.1016/j.spinee.2020.06.022

[12] Shen F, Kim HJ, Lee NK, Chun HJ, Chang BS, Lee CK, et al. The influence of hand grip strength on surgical outcomes after surgery for degenerative lumbar spinal stenosis: a preliminary result. Spine J. 2018;18(11):2018–24. 10.1016/j.spinee.2018.04.009 29679727 10.1016/j.spinee.2018.04.009

[13] Kwon O, Kim HJ, Shen F, Park SM, Chang BS, Lee CK, et al. Influence of hand grip strength on surgical outcomes after surgery for adult spinal deformity. Spine (Phila Pa 1976). 2020;45(22):E1493–9. 10.1097/BRS.0000000000003636 32756282 10.1097/BRS.0000000000003636

[14] Ballinger GA. Organizational Res Methods. 2004;7(2):127–50. 10.1177/1094428104263672. Using Generalized Estimating Equations for Longitudinal Data Analysis.

[15] Twisk JW. Longitudinal data analysis. A comparison between generalized estimating equations and random coefficient analysis. Eur J Epidemiol. 2004;19(8):769–. 10.1023/b:ejep.0000036572.00663.f2. 76. 15469034 10.1023/b:ejep.0000036572.00663.f2

[16] Garg B, Mehta N. Minimally invasive transforaminal lumbar interbody fusion (MI-TLIF): A review of indications, technique, results and complications. J Clin Orthop Trauma. 2019;10(Suppl 1):S156–62. 10.1016/j.jcot.2019.01.008 31695275 10.1016/j.jcot.2019.01.008 PMC6823784

[17] Wang CY, Olson SL, Protas EJ. Test-retest strength reliability: hand-held dynamometry in community-dwelling elderly fallers. Arch Phys Med Rehabil. 2002;83(6):811–5. 10.1053/apmr.2002.32743 12048660 10.1053/apmr.2002.32743

[18] Fang X, Zhang M, Wang L, Hao Z. Comparison of PLIF and TLIF in the treatment of LDH complicated with spinal stenosis. J Healthc Eng. 2022;2022:9743283. 10.1155/2022/9743283 35378938 10.1155/2022/9743283 PMC8976646

[19] Fujimori T, Okuda S, Iwasaki M, Yamasaki R, Maeno T, Yamashita T, et al. Validity of the Japanese orthopaedic association scoring system based on patient-reported improvement after posterior lumbar interbody fusion. Spine J. 2016;16(6):728–36. 10.1016/j.spinee.2016.01.181 26826003 10.1016/j.spinee.2016.01.181

[20] Lee HY, Hung MC, Hu FC, Chang YY, Hsieh CL, Wang JD. Estimating quality weights for EQ-5D (EuroQol-5 dimensions) health States with the time trade-off method in Taiwan. J Formos Med Association = Taiwan Yi Zhi. 2013;112(11):699–706. 10.1016/j.jfma.2012.12.015 10.1016/j.jfma.2012.12.015 24183199

[21] Wade DT, Collin C. The Barthel ADL index: a standard measure of physical disability? Int Disabil Stud. 1988;10(2):64–7. 10.3109/09638288809164105 3042746 10.3109/09638288809164105

[22] Browner WSNT, Hulley SB. Appendix 6 C: Total Sample Size Required When Using the Correlation Coefficient (r). Designing clinical research: an epidemiologic approach. Philadelphia: Lippincott Williams & Wilkins; 2013;79.

[23] Arunakul R, Anumas S, Pattharanitima P, Susrivaraput C, Pholsawatchai W. Unilateral biportal endoscopic versus microscopic transforaminal lumbar interbody fusion for lumbar degenerative disease: a retrospective study. J Orthop Surg Res. 2024;19(1):326. 10.1186/s13018-024-04813-w 38824551 10.1186/s13018-024-04813-w PMC11144317

[24] Wang W, Cui Y, Sun X, Zhang H, Yin W, Cui X, et al. Transforaminal posterior lumbar interbody fusion microscopic safe operating area: a three-dimensional model study based on computed tomography imaging. J Orthop Surg Res. 2024;19(1):342. 10.1186/s13018-024-04830-9 38849945 10.1186/s13018-024-04830-9 PMC11161984

[25] Guo W, Ye J, Li T, Yu Y, Fan X. Evaluation of the learning curve and complications in unilateral biportal endoscopic transforaminal lumbar interbody fusion: cumulative sum analysis and risk-adjusted cumulative sum analysis. J Orthop Surg Res. 2024;19(1):194. 10.1186/s13018-024-04674-3 38509573 10.1186/s13018-024-04674-3 PMC10956305

[26] Massy-Westropp NM, Gill TK, Taylor AW, Bohannon RW, Hill CL. Hand grip strength: age and gender stratified normative data in a population-based study. BMC Res Notes. 2011;4:127. 10.1186/1756-0500-4-127 21492469 10.1186/1756-0500-4-127 PMC3101655

[27] Fielding RA, Sarcopenia. An emerging syndrome of advancing age. Calcif Tissue Int. 2024;114(1):1–2. 10.1007/s00223-023-01175-z 38189814 10.1007/s00223-023-01175-z

[28] Tay L, Ding YY, Leung BP, Ismail NH, Yeo A, Yew S, et al. Sex-specific differences in risk factors for sarcopenia amongst community-dwelling older adults. Age (Dordr). 2015;37(6):121. 10.1007/s11357-015-9860-3 26607157 10.1007/s11357-015-9860-3 PMC5005859

[29] Alswat KA. Gender disparities in osteoporosis. J Clin Med Res. 2017;9(5):382–7. 10.14740/jocmr2970w 28392857 10.14740/jocmr2970w PMC5380170

[30] Soraya N, Parwanto E. The controversial relationship between body mass index and handgrip strength in the elderly: an overview. Malays J Med Sci. 2023;30(3):73–83. 10.21315/mjms2023.30.3.6 37425376 10.21315/mjms2023.30.3.6 PMC10325128

[31] Ko SH, Park SJ, Kim NY, Jeon W, Shin DA, Kim SH. Influence of preoperative handgrip strength on length of stay after lumbar fusion surgery. J Clin Med. 2022;11(14). 10.3390/jcm11143928 10.3390/jcm11143928 PMC9323344 35887694

[32] Liu YC, Huang SW, Adams CR, Lin CY, Chen YP, Kuo YJ, et al. Preoperative handgrip strength can predict early postoperative shoulder function in patients undergoing arthroscopic rotator cuff repair. J Orthop Surg Res. 2024;19(1):270. 10.1186/s13018-024-04750-8 38689328 10.1186/s13018-024-04750-8 PMC11059705

[33] Chen CH, Ho C, Huang YZ, Hung TT. Hand-grip strength is a simple and effective outcome predictor in esophageal cancer following esophagectomy with reconstruction: a prospective study. J Cardiothorac Surg. 2011;6:98. 10.1186/1749-8090-6-98 21843340 10.1186/1749-8090-6-98 PMC3170319

[34] Han J, Kim CH, Kim JW. Handgrip strength effectiveness and optimal measurement timing for predicting functional outcomes of a geriatric hip fracture. Sci Rep. 2022;12(1):20600. 10.1038/s41598-022-25177-3 36446812 10.1038/s41598-022-25177-3 PMC9708680

[35] Chen YP, Wong PK, Tsai MJ, Chang WC, Hsieh TS, Leu TH, et al. The high prevalence of sarcopenia and its associated outcomes following hip surgery in Taiwanese geriatric patients with a hip fracture. J Formos Med Association = Taiwan Yi Zhi. 2020;119(12):1807–16. 10.1016/j.jfma.2020.02.004 32107098 10.1016/j.jfma.2020.02.004

[36] Norman K, Stobaus N, Gonzalez MC, Schulzke JD, Pirlich M. Hand grip strength: outcome predictor and marker of nutritional status. Clin Nutr. 2011;30(2):135–42. 10.1016/j.clnu.2010.09.010 21035927 10.1016/j.clnu.2010.09.010

[37] MacLean MA, Touchette CJ, Han JH, Christie SD, Pickett GE. Gender differences in the surgical management of lumbar degenerative disease: a scoping review. J Neurosurg Spine. 2020;32(6):799–816. 10.3171/2019.11.SPINE19896 32005013 10.3171/2019.11.SPINE19896

[38] Salamanna F, Contartese D, Tschon M, Borsari V, Griffoni C, Gasbarrini A, et al. Sex and gender determinants following spinal fusion surgery: A systematic review of clinical data. Front Surg. 2022;9:983931. 10.3389/fsurg.2022.983931 36325040 10.3389/fsurg.2022.983931 PMC9618873

[39] Bizzoca D, Solarino G, Pulcrano A, Brunetti G, Moretti AM, Moretti L, et al. Gender-Related issues in the management of Low-Back pain: A current concepts review. Clin Pract. 2023;13(6):1360–8. 10.3390/clinpract13060122 37987423 10.3390/clinpract13060122 PMC10660510

[40] Bartley EJ, Fillingim RB. Sex differences in pain: a brief review of clinical and experimental findings. Br J Anaesth. 2013;111(1):52–8. 10.1093/bja/aet127 23794645 10.1093/bja/aet127 PMC3690315

[41] Failla MD, Beach PA, Atalla S, Dietrich MS, Bruehl S, Cowan RL, et al. Gender differences in pain threshold, unpleasantness, and descending pain modulatory activation across the adult life span: A cross sectional study. J Pain. 2024;25(4):1059–69. 10.1016/j.jpain.2023.10.027 37956742 10.1016/j.jpain.2023.10.027 PMC10960699

[42] Nakajima K, Miyahara J, Ohtomo N, Nagata K, Kato S, Doi T, et al. Impact of body mass index on outcomes after lumbar spine surgery. Sci Rep. 2023;13(1):7862. 10.1038/s41598-023-35008-8 37188788 10.1038/s41598-023-35008-8 PMC10185565

[43] Bono OJ, Poorman GW, Foster N, Jalai CM, Horn SR, Oren J, et al. Body mass index predicts risk of complications in lumbar spine surgery based on surgical invasiveness. Spine J. 2018;18(7):1204–10. 10.1016/j.spinee.2017.11.015 29155339 10.1016/j.spinee.2017.11.015

[44] Hirahata M, Yasui Y, Fujita M, Ishii K, Kawano H, Kitagawa T. Overweight increases perioperative spinal surgery complications: a single-center retrospective study. BMC Musculoskelet Disord. 2023;24(1):98. 10.1186/s12891-023-06217-z 36740675 10.1186/s12891-023-06217-z PMC9900974

[45] Olson TE, Upfill-Brown A, Adejuyigbe B, Bhatia N, Lee YP, Hashmi S, et al. Does obesity and varying body mass index affect the clinical outcomes and safety of biportal endoscopic lumbar decompression? A comparative cohort study. Acta Neurochir (Wien). 2024;166(1):246. 10.1007/s00701-024-06110-1 38831229 10.1007/s00701-024-06110-1 PMC11147858

[46] Cofano F, Perna GD, Bongiovanni D, Roscigno V, Baldassarre BM, Petrone S, et al. Obesity and spine surgery: A qualitative review about outcomes and complications. Is it time for new perspectives on future researches?? Global Spine J. 2022;12(6):1214–30. 10.1177/21925682211022313 34128419 10.1177/21925682211022313 PMC9210241

[47] Xu YZ, Wang YT, Fan P, Yin XJ, Liu H, Jiang F. Complications and outcomes of open posterior lumbar spinal fusion surgery in obese patients: a meta-analysis. Br J Neurosurg. 2022;36(4):427–35. 10.1080/02688697.2020.1867059 33377806 10.1080/02688697.2020.1867059

[48] Goyal A, Elminawy M, Kerezoudis P, Lu VM, Yolcu Y, Alvi MA, et al. Impact of obesity on outcomes following lumbar spine surgery: A systematic review and meta-analysis. Clin Neurol Neurosurg. 2019;177:27–36. 10.1016/j.clineuro.2018.12.012 30583093 10.1016/j.clineuro.2018.12.012

[49] Shinonara K, Ugawa R, Arataki S, Nakahara S, Takeuchi K. Charlson comorbidity index is predictive of postoperative clinical outcome after single-level posterior lumbar interbody fusion surgery. J Orthop Surg Res. 2021;16(1):235. 10.1186/s13018-021-02377-7 33785033 10.1186/s13018-021-02377-7 PMC8008557

[50] Whitmore RG, Stephen JH, Vernick C, Campbell PG, Yadla S, Ghobrial GM, et al. ASA grade and Charlson comorbidity index of spinal surgery patients: correlation with complications and societal costs. Spine J. 2014;14(1):31–8. 10.1016/j.spinee.2013.03.011 23602377 10.1016/j.spinee.2013.03.011

[51] McGee A, Levitt EB, Prather JC, Crowther D, McGwin G, Theiss S, et al. Association of mortality and Charlson comorbidity index in surgical spinal trauma patients at a level I academic center. J Am Acad Orthop Surg. 2022;30(5):215–22. 10.5435/JAAOS-D-21-00916 35050938 10.5435/JAAOS-D-21-00916

[52] Cha EDK, Lynch CP, Jadczak CN, Mohan S, Geoghegan CE, Singh K. Comorbidity influence on postoperative outcomes following anterior cervical discectomy and fusion. Neurospine. 2021;18(2):271–80. 10.14245/ns.2040646.323 34218609 10.14245/ns.2040646.323 PMC8255775

[53] Bays A, Stieger A, Held U, Hofer LJ, Rasmussen-Barr E, Brunner F, et al. The influence of comorbidities on the treatment outcome in symptomatic lumbar spinal stenosis: A systematic review and meta-analysis. N Am Spine Soc J. 2021;6:100072. 10.1016/j.xnsj.2021.100072 35141637 10.1016/j.xnsj.2021.100072 PMC8820012

